# miR-200b downregulates CFTR during hypoxia in human lung epithelial cells

**DOI:** 10.1186/s11658-017-0054-0

**Published:** 2017-11-15

**Authors:** Sylwia Bartoszewska, Wojciech Kamysz, Bogdan Jakiela, Marek Sanak, Jarosław Króliczewski, Zsuzsa Bebok, Rafal Bartoszewski, James F. Collawn

**Affiliations:** 1 Department of Inorganic Chemistry, Medical University of Gdansk, Gdansk, Poland; 2 Department of Medicine, Jagiellonian University Medical College, Krakow, Poland; 3 Department of Chemical Biology, Faculty of Biotechnology, University of Wroclaw, Wroclaw, Poland; 4 Department of Cell, Developmental and Integrative Biology, University of Alabama at Birmingham, Birmingham, USA; 5 Department of Biology and Pharmaceutical Botany, Medical University of Gdansk, Hallera 107, 80-416 Gdansk, Poland

**Keywords:** CFTR, HIF-1, micro-RNA 200b, hsa-miR-200b-3p

## Abstract

**Background:**

Hypoxic conditions induce the expression of hypoxia-inducible factors (HIFs) that allow cells to adapt to the changing conditions and alter the expression of a number of genes including the cystic fibrosis transmembrane conductance regulator (CFTR). *CFTR* is a low abundance mRNA in airway epithelial cells even during normoxic conditions, but during hypoxia its mRNA expression decreases even further.

**Methods:**

In the current studies, we examined the kinetics of hypoxia-induced changes in *CFTR* mRNA and protein levels in two human airway epithelial cell lines, Calu-3 and 16HBE14o-, and in normal primary bronchial epithelial cells. Our goal was to examine the posttranscriptional modifications that affected CFTR expression during hypoxia. We utilized in silico predictive protocols to establish potential miRNAs that could potentially regulate *CFTR* message stability and identified miR-200b as a candidate molecule.

**Results:**

Analysis of each of the epithelial cell types during prolonged hypoxia revealed that *CFTR* expression decreased after 12 h during a time when miR-200b was continuously upregulated. Furthermore, manipulation of the miRNA levels during normoxia and hypoxia using miR-200b mimics and antagomirs decreased and increased *CFTR* mRNA levels, respectively, and thus established that miR-200b downregulates *CFTR* message levels during hypoxic conditions.

**Conclusion:**

The data suggest that miR-200b may be a suitable target for modulating CFTR levels in vivo.

**Electronic supplementary material:**

The online version of this article (10.1186/s11658-017-0054-0) contains supplementary material, which is available to authorized users.

## Background

Cystic fibrosis is a lethal monogenic disease caused by mutations in the cystic fibrosis transmembrane conductance regulator (CFTR) [1]. The CFTR protein is a chloride-bicarbonate channel that is expressed at low levels in epithelial cells of the airway, and at higher levels in epithelial cells in the intestine, pancreatic duct and male genital duct [2]. The post-transcriptional regulation of *CFTR* expression is controlled, at least in part, by microRNAs and this type of regulation has been demonstrated in Caco-2 cells, a human colon carcinoma cell line [3]. Studies by Gillen et al. [3] show that five microRNAs repress endogenous CFTR expression in this cell line, supporting the hypothesis that differences in the miRNA profiles in various tissues modulate the expression of *CFTR* to different degrees.

In a transcriptomic mRNA and miRNA array-based analysis of the human colonic epithelial cell line HT29, Guimbellot and colleagues demonstrated that hypoxia mimetics treatment decreased *CFTR* message levels and that a number of miRNAs were upregulated [4]. Other studies have shown that miRNAs play a role in the posttranscriptional regulation of *CFTR* expression for both the wild-type protein and the most common mutation in cystic fibrosis, ΔF508 CFTR [5]. miRNAs are endogenous single-stranded RNAs that regulate the expression of specific genes at the posttranscriptional level [6, 7]. They regulate gene expression by binding to a specific sequence in the 3′UTR or sometimes 5′UTR of a target mRNA [8, 9].

Previous studies have shown that some miRNAs are induced during hypoxia and play a critical role in the cellular adaptive response to low oxygen levels [10]. Using in silico analysis (miRANDA and TargetScan algorithms) of miRNAs induced during hypoxia, we identified miR-200b as a potential novel regulator of *CFTR* mRNA levels. Experimental validation was confirmed in two human epithelial cell lines and in human primary lung epithelial cells and the results indicate that during hypoxia, miR-200b decreases *CFTR* mRNA levels in a time-course dependent manner.

## Methods

### Cell lines and culture conditions

Calu3 (ATCC® HTB-55™) and HEK293 (ATCC® CRL-1573) cells were obtained from ATCC. 16HBE14o- cells and HeLaWT were obtained as previously described [11, 12]. Cells were cultured in Minimum Essential modified Eagle’s medium (Invitrogen) with 10% fetal bovine serum in a humidified incubator at 37 °C in 5% CO_2_ in 6-well plates and allowed to grow to 70–80% confluence prior to the start of the experiments.

Primary human bronchial epithelial cells (NHBEC) were derived from brushings of bronchial mucosa obtained during bronchoscopy in normal individuals (i.e., patients referred for diagnostic bronchoscopy in which chronic airway disease was excluded during the further clinical investigation), and aged 30–64 (all donors were current non-smokers). NHBEC were isolated by enzymatic digestion (pronase and DNAse I, Sigma-Aldrich, St. Louis, MO), cultured in supplemented bronchial epithelial growth medium (BEGM; Lonza, Basel, Switzerland) until confluent, and cryopreserved (passage 1) for further experiments. The sampling protocol was approved by Jagiellonian University Bioethics Committee, and informed consent was obtained from all participants. For experiments, thawed primary NHBEC were grown in BEGM medium (Lonza), as an adherent cell line, and maintained in culture until passage 5. Cells were seeded into 6-well plates or 2 cm dishes and allowed to grow to 70–80% confluence prior to the start of the experiments.

### Induction of hypoxia

Hypoxia was induced in a CO_2_/O_2_ incubator/chamber for hypoxia research (Invivo2, Baker Ruskin). Briefly, cells were cultured in 2 cm dishes at 0.9% O_2_ for the time periods specified. Control cells were maintained in normoxic conditions in the same incubator and harvested at the specified times.

### Isolation of RNA and microRNA

Total RNA containing the microRNA fraction was isolated using the miRNeasy kit (Qiagen). RNA concentrations were calculated based on the absorbance at 260 nm. RNA samples were stored at −70 °C until use.

### 5′UTR and 3′UTR CFTR Luciferase reporter assays

A human 5′UTR *CFTR* promoter-driven firefly luciferase reporter construct (p*CFTR*-pLuc) was purchased from Panomics (Cat. #: LR1020, Panomics Inc., Fremont, CA). This construct contains a 1000-bp fragment of the human *CFTR* 5′-UTR upstream of firefly luciferase as described in [13]. A human 3′UTR *CFTR* firefly luciferase reporter construct was purchased from GeneCopoeia (HmiT000948-MT06 - miRNA 3′UTR target expression clone for NM_000492.3) along with the control vector (CmiT000001-MT06 (miRNA Target clone control vector for pEZX-MT06).

To test the transcriptional and post-transcriptional activity of the human *CFTR* UTR regions, Calu3 cells and HEK293 were transfected with the constructs described above or with control plasmids provided by Panomics/GeneCopoeia. Twenty-four hours before experiments, cells were seeded onto 6-well plates at ∼40% confluency and transfected using Lipofectamine 2000 (Invitrogen). For the specified experiments, miR-200b and miR-200c analogs were cotransfected. Each well received 2 μg of total plasmid DNA and 1 μg of a vector of interest plus 1 μg of *Renilla* luciferase as an internal control for the 5′UTR or 3′UTR constructs. For the 5′UTR and 3′UTR cotransfections (2 μg total), 1 μg of each reporter vector was used as well as 1 μg of *Renilla* luciferase as an internal control. miR-200b mimic was used at final concentration of 10 nM. At the time points indicated, cells were lysed using luciferase assay lysis buffer (Promega) and firefly/*Renilla* luciferase activities were measured using the Dual-Luciferase Reporter Assay (Promega) according to the manufacturer’s protocol. Results in treated cells were plotted as the percent decrease in arbitrary light units compared with control cells.

### Measurement of mRNA and miRNA levels using quantitative Real Time PCR (qRT-PCR)

We used TaqManOne-Step RT-PCR Master MixReagents (Applied Biosystems) as described previously [14, 15] using the manufacturer’s protocol. The relative expressions were calculated using the comparative relative standard curve method [16]. We used *18S* rRNA as the relative control for our studies. We also validated this relative control against another housekeeping gene, TATA-binding protein (*TBP*). As relative controls for miRNA quantification, we validated and used *RNU48*. TaqMan probes ids used were: *CFTR -* Hs00357011_m1; *18S* - Hs99999901_s1; *TBP* - Hs4332659_m1; *RNU48* – 001006; miR-200b – 002251; miR-200c - 002300.

### miRNA analog transfections

miR-200b mimic (id MC10492) and antagomiR (id MH10492), as well as miR-200c mimic (id MC11714) and antagomiR (id MH11714), were purchased from Ambion. Cells were transfected using the Lipofectamine RNAiMax according to manufacturer’s protocol. miR-200b/c mimics and antagomiRs were used at final concentrations of 10 nM and 20 nM, respectively. The transfected cells were cultured for 2 days prior to further analysis. The degree of miRNA over-expression or knockdown was determined by qRT-PCR (Additional file 1: Figure S1B). cel-miR-67 was used as a control (Ambion assay id MC22484). As additional controls, Ambion siRNA Negative Control 1 (no. 4390843), Ambion mimic control (no. 4464060) and Ambion antagomiR control (no. 4464076) were used as well.

### Western blots

Cells were lysed in RIPA buffer (150 mM NaCl, 1% NP-40, 0.5% sodium deoxycholate, 0.1% SDS, 50 mM Tris- HCl, pH 8.0) supplemented with protease Inhibitor Complete Mini (Roche) on ice for 15 min. The cell lysates were rotated at 4 °C for 30 min and the insoluble material was removed by centrifugation at 15,000 g for 15 min. Protein concentrations were determined by BioRad™ Protein Assay using bovine serum albumin (BSA) as a standard. Following the normalization of protein concentrations, lysates were mixed with an equal volume of 2X Laemmli sample buffer and incubated for 5 min at 95 °C prior to separation by SDS PAGE on stain-free TGX gradient gels (BioRad). Following SDS-PAGE, the proteins were transferred to polyvinylidene fluoride membranes (300 mA for 90 min at 4 °C). The membranes were then blocked with BSA (Sigma-Aldrich) dissolved in PBS/Tween-20 (3% BSA, 0.5% Tween-20 for 1–2 h), followed by immunoblotting with the primary antibody specified for each experiment CFTR (Merck MM13–4 diluted at 1:1000), HIF-1α (Abcam ab16066, diluted at 1:1000); and beta ACTIN (Abcam ab1801, diluted at 1:1000). After the washing steps, the membranes were incubated with goat anti-rabbit IgG (H + L chains) or with goat anti-mouse IgG (H + L) HRP-conjugated secondary antibodies (BioRad) and detected using ECL (Amresco). Densitometry was performed using Image Lab software v. 4.1 (BioRad).

### Statistical analysis

The results were expressed as the mean ± standard deviation (SD). Statistical significance among means was determined using the Student’s t-test (two samples, paired and unpaired) [17]. Analyzes were performed with Dell Statistica version 13 (Dell Inc., 2016).

## Results

### Downregulation of CFTR during hypoxia involves post-transcriptional HIF-1-dependent mechanisms

**Fig. 1:**
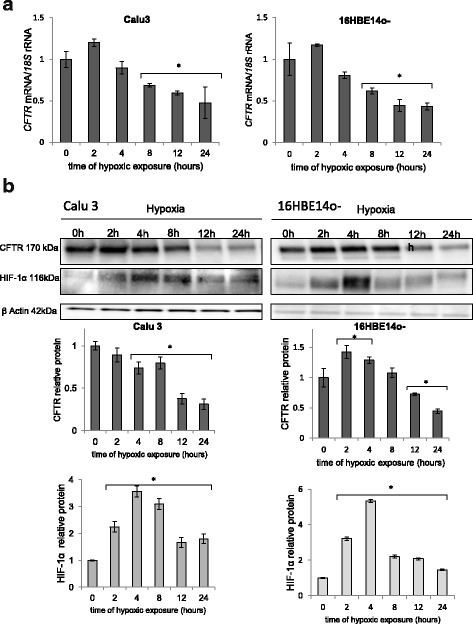
Regulation of *CFTR* during hypoxia in human lung epithelial cells, Calu3 cells (left panels) and 16HBE14o- cells (right panels). **a** *CFTR* mRNA is reduced during hypoxia. *CFTR* mRNA levels were monitored in qRT-PCR experiments. The results from 3 independent experiments (*n* = 12) are plotted normalized to 18S rRNA levels and expressed as a fold-change over the normoxic control. **b** Hypoxia sequentially increases HIF-1α protein levels and reduces CFTR protein levels. Protein expression levels of were monitored with SDS-PAGE and Western Blot and normalized to β-actin levels. Two individual samples (4 μg of total protein per lane) were tested for each time point and the experiments were repeated twice. Error bars represent standard deviations. Significant changes (*P* < 0.05) are marked with an asterisk

Previous studies reported that CFTR protein and mRNA levels were reduced during hypoxia in human lung epithelial cells [18, 19]. However, these reports were limited to chemical hypoxia induction and a single time point of physiological hypoxia and therefore did not provide the information about the dynamics of hypoxic *CFTR* message downregulation. Hence, to examine the kinetics of hypoxia-induced changes of CFTR protein and mRNA levels, we performed time-course studies during physiological hypoxia in two lung epithelial cell lines, Calu3 cells (epithelial lung adenocarcinoma; derived from metastatic site: pleural effusion) and 16HBE14o- cells (SV40-immortalized human bronchial epithelial cells). Both Calu3 and 16HBE14o- cell lines are commonly used in vitro for studying CFTR biogenesis and function. As shown in Fig. 1a, the changes in *CFTR* mRNA profiles show a significant decrease in mRNA after 8 h in both cell lines and correlate well with the CFTR protein changes (Fig. 1b). During the early stages of hypoxia up to 8 h, CFTR protein levels remain either mostly unchanged (Calu3 cells) or slightly induced (16HBE14o- cells), whereas, under chronic hypoxia (after 12 h), CFTR levels are significantly reduced and drop below half of the normoxic levels in both cell lines.

**Fig. 2:**
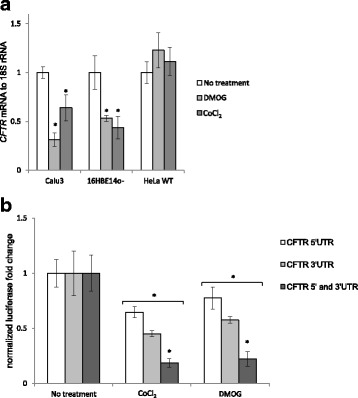
Downregulation of *CFTR* expression during hypoxia is HIF-1 dependent and relies on both the 5′ and 3′ UTRs of *CFTR* mRNA. **a** Calu3, 16HBE14o- and HeLa WT CFTR cells were treated with hypoxia mimetics (500 μM DMOG for 12 h (light grey) and 200 μM CoCl_2_ for 12 h (dark grey)) and the mRNA levels were monitored in qRT-PCR experiments. *CFTR* mRNA levels from 2 independent experiments (*n* = 8) are plotted relative to *18S rRNA* levels and expressed as a fold change over the untreated control. **b** Calu3 cells were transfected with 5′UTR *CFTR* luciferase reporter (white), 3′UTR *CFTR* luciferase reporter (light grey) and co-transfected with both 5′UTR and 3′UTR *CFTR* luciferase reporters (dark grey) and treated with hypoxia mimetics (500 μM DMOG or 200 μM CoCl_2_ for 12 h) and the luciferase activity was monitored. These reporters were normalized to internal controls (*Renilla*) firefly luciferase activities from 2 independent experiments (*n* = 6) and plotted and expressed as a fold-change over non-treated control. Error bars represent standard deviations. Significant changes (*P* < 0.05) are marked with an asterisk

In a previous study, Zheng and coworkers reported that HIF-1 is responsible for decreases in *CFTR* mRNA and protein [18], and therefore we followed the levels *HIF1A* message and HIF-1α protein during the hypoxia time course as well (Fig. 1a, b). In both Calu3 and 16HBE14o- cells, the maximum of HIF-1α protein increases during hypoxia immediately preceded the decline in CFTR protein and mRNA, which is consistent with previous studies in intestinal epithelia that HIF-1 decreases *CFTR* mRNA levels during hypoxia. To test this hypothesis using another approach, we utilized hypoxia mimetics (CoCl_2_ and dimethyloxalylglycine (DMOG)) that stabilize the HIF-1α protein and thus induce *HIF-1* transcriptional activity [20]. We analyzed the related changes in *CFTR* mRNA in Calu3 and 16HBE14o- cells as well as in a HeLa cell line that expresses recombinant *CFTR* mRNA (HeLaWT) that does not contain the 5′ or 3′ UTRs of *CFTR* (Fig. 2a). The chemically stabilized HIF-1 activity mediated by the hypoxia mimetics decreased *CFTR* mRNA in both Calu3 and16HBE14o- cells, whereas it had no effect on exogenous *CFTR* mRNA levels in HeLaWT cells.

While these data confirm that during hypoxia, *CFTR* mRNA is decreased, they do not address whether this is a transcriptional and/or post-transcriptional HIF-1-dependent mechanism. Since we did not observe a clear negative correlation between *CFTR* mRNA and HIF-1α expression profiles (Fig. 1), this suggested the possibility that other HIF-1-dependent secondary post-transcriptional factors might be responsible for the decline in *CFTR* message and protein expression. To examine this hypothesis, we tested the function of the *CFTR* 5′UTR and 3′UTR mRNAs during hypoxia using specific luciferase reporters. As shown in Fig. 2b, the hypoxia mimetics CoCl_2_ and DMOG reduced both 5′ UTR- and 3′UTR-dependent luciferase expression, suggesting that both untranslated regions of *CFTR* mRNA are involved in HIF-1-dependent reduction of *CFTR* mRNA. Interestingly, luciferase expression from the *CFTR* 3′UTR reporter construct was more inhibited than the 5′UTR reporter, and that the two effects were additive. This suggested a synergistic effect of both UTRs in reducing *CFTR* mRNA during hypoxia and the involvement of both the 5′UTR and 3′UTR in transcriptional/post-transcriptional HIF-1-dependent mechanisms.

### miR-200b is induced by hypoxia in a HIF-1-dependent manner in human lung epithelial cells

**Fig. 3:**
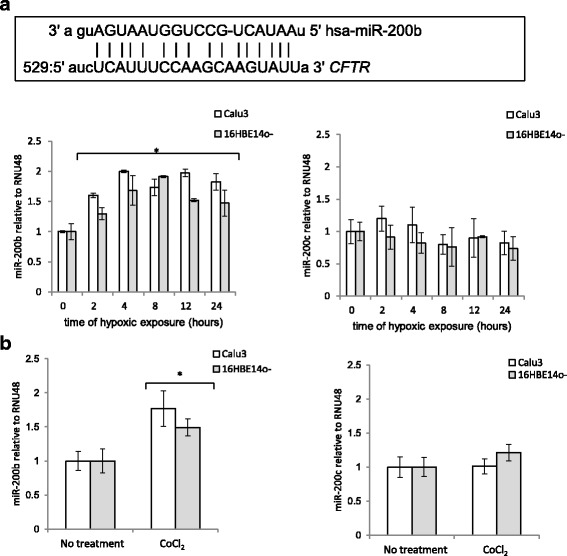
Hypoxia induces miR-200b in human airway epithelial cells in a HIF-1-dependent manner. **a** The predicted target site of miR-200b in *CFTR* 3′UTR is shown above. The miR-200b target site was predicted in human *CFTR* 3′UTR only, Hypoxia-induced changes in the expression profiles of miR-200b and miR-200c in Calu3 and 16HBE14o- cells are shown. The miRNA levels were monitored in qRT-PCR experiments. The results from 2 independent experiments (*n* = 8) are plotted normalized to *RNU48* and expressed as a fold-change over the normoxic control. **b** Calu3 and 16HBE14o- cells were treated with hypoxia mimetic (200 μM CoCl_2_ for 12 h) and the miRNA levels were monitored in qRT-PCR experiments. miR-200b and miR-200c levels were measured in 3 independent experiments (*n* = 10) and are plotted relative to *RNU44* levels and expressed as a fold change over the untreated controls. Error bars represent standard deviations (SD). Significant changes (*P* < 0.05) are marked with an asterisk

The expression of many miRNAs has been shown to be HIF-1 dependent under hypoxia [21]. To test the hypothesis that such a HIF-1-dependent miRNA could contribute to *CFTR* downregulation, we analyzed the *CFTR* 3′UTR sequence for potential binding sites using the miRANDA and TargetScan algorithms [22, 23]. Using this approach, we identified a potential target site for miR-200b/200c at position 529 bases from the stop codon in the 3′UTR of *CFTR* mRNA (Fig. 3). Since the expression of miR-200b and miR-200c was previously reported to be hypoxia-dependent in human endothelial cells, we tested their expression profiles during hypoxia in the Calu3 and 16HBE14o- cells. As shown in Fig. 3a, miR-200b was induced up to 2-fold during the hypoxia time course in both cell lines, whereas miR-200c was not elevated and therefore probably not involved in CFTR regulation during hypoxia. The miR-200b levels were elevated to a maximum level at 4 h and that correlated well with HIF-1’s maximal expression, and importantly, miR-200b levels remained elevated throughout the 24-h test period. Furthermore, the increase in miR-200b levels correlated negatively with the respective decrease in *CFTR* mRNA and protein, and supported miR-200b’s role in regulating CFTR expression.

Although in our previous studies in human primary endothelial cells (HUVECs), we identified a HIF response element (HRE) consensus in the proximity of miR-200b sequence, the hypoxia mimetics had limited impact on miR-200b’s expression [24]. However, miRNA expression, as well as HIF-1 activity, is often tissue-specific, and therefore we tested whether the induction of HIF-1 activity would affect miR-200b expression in lung epithelial cells. As shown in Fig. 3b, CoCl_2_ induced HIF-1 activity and resulted in the elevation of miR-200b levels in both Calu3 and 16HBE14o- cells, suggesting that hypoxic induction of this miRNA is HIF-1 dependent. Furthermore, CoCl_2_ treatment had no significant effect on miR-200c expression.

### miR-200b binds to CFTR’s 3′UTR

**Fig. 4:**
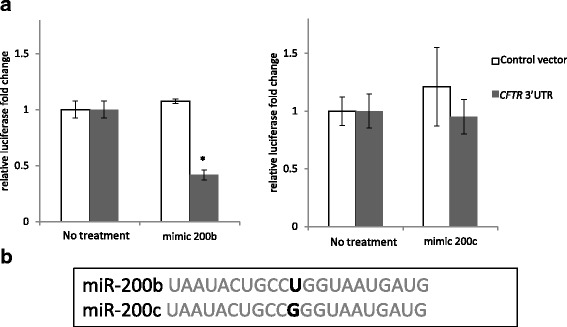
miR-200b binds to the predicted target sequence in the *CFTR* 3′UTR. **a** HEK293 cells were transfected with 3′UTR *CFTR* luciferase reporter construct alone (white) or together with miR-200b mimic (grey, left panel) or miR-200c mimic (grey, right panel). Similar experiments were performed on control vector that did not contain the miR-200b/miR-200c target site (not shown). Data were normalized to control *Renilla* luciferase activities from 2 independent experiments (*n* = 6) and are expressed as a fold-change over control. Error bars represent standard deviations (SD). Significant changes (*P* < 0.05) are marked with an asterisk. **b** The comparison of miR-200b and miR-200c seed sequences is shown

Although the miRNAs recognize specific target sequences, these sequences (6–8 nt) can be present in the 3′UTRs of many different genes. Hence, in order to exclude indirect effects of miR-200b on CFTR expression, we utilized 3′UTR luciferase reporter. Briefly, a plasmid containing the 3′ UTR of human *CFTR* gene was tested in a luciferase gene construct that was co-expressed in human embryonic kidney cells 293 (HEK293) in the presence and absence of a miR-200b analog (mimic). HEK293 cells were used since they express very low endogenous levels of miR-200b/c. As shown in Fig. 4a, miR-200b overexpression resulted in significantly reduced luciferase expression compared to the no treatment control. Furthermore, a similar experiment with miR-200c that has only one base difference in seed sequence from miR-200b did not result in a luciferase signal reduction (Fig. 4b), confirming the direct interaction between miR-200b and its target site at 3′UTR of *CFTR* mRNA.

**Fig. 5:**
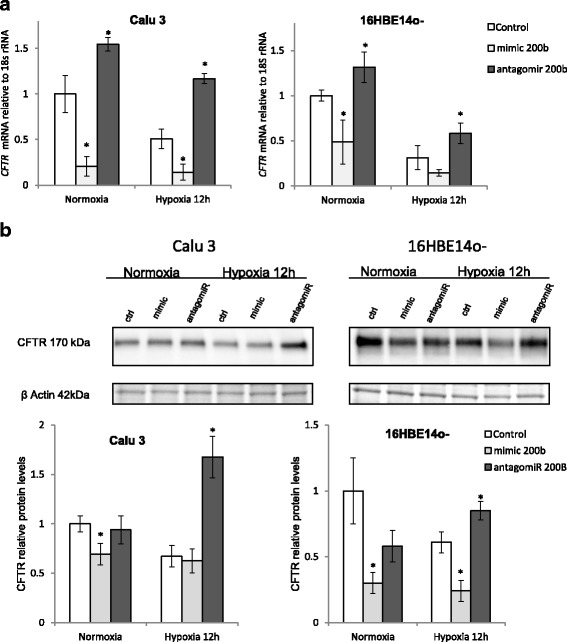
miR-200b decreases the expression of *CFTR* mRNA during normoxia and hypoxia. **a** Calu3 and 16HBE14o- cells were transfected with miR-200b mimic or antagomir and the mRNA levels were monitored in qRT-PCR experiments in normoxic conditions and after 12 h of hypoxia. *CFTR* mRNA levels from 2 independent experiments (*n* = 8) are normalized to 18S rRNA levels and expressed as a fold change over the transfection control. **b** The corresponding changes in CFTR protein levels of were detected with SDS-PAGE and Western Blot analyses and normalized to the β-actin levels. Two individual samples (3 μg of total protein per lane) were tested for each treatment and the experiments were repeated twice. Error bars represent standard deviations (SD). Significant changes (*P* < 0.05) are marked with an asterisk

Next, we tested the effects of miR-200b overexpression and inhibition on *CFTR* mRNA levels after 12 h of hypoxia. The miR-200b upregulation with mimic reduced *CFTR* mRNA in hypoxia and normoxia in both Calu3 and 16HBE14o- (Fig. 5a). Furthermore, the inhibition of miR-200b activity with antagomiR increased *CFTR* mRNA in both cell lines (Fig. 5a). In parallel, we followed miR-200b analogs effect on CFTR protein levels. As shown in Fig. 5b, in normoxia and during hypoxia, miR-200b overexpression resulted in the reduction of CFTR protein levels in 16HBE14o- cells. Whereas in Calu3 cells, mimic only had an effect during normoxia, although both cell lines had elevated CFTR protein with the antagomir treatment during hypoxia, confirming the physiological effect of miR-200b CFTR expression during low oxygen levels.

**Fig. 6:**
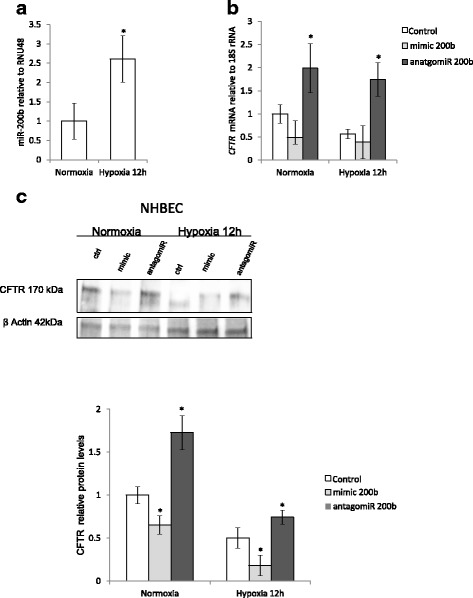
miR-200b decreases the expression of *CFTR* mRNA during normoxia and hypoxia in primary normal human bronchial epithelial cells (NHBEC). **a** The levels of miR-200b after 12 h of hypoxia in NHBECs from 3 independent experiments (*n* = 6) are plotted normalized to *RNU44* levels and expressed as a fold change over the normoxia control. **b** NHBEC cells were transfected with miR-200b mimic or antagomir and the mRNA levels were monitored in qRT-PCR experiments in normoxic conditions and after 12 h of hypoxia. *CFTR* mRNA levels from 2 independent experiments (*n* = 8) are plotted normalized to 18S rRNA levels and expressed as a fold change over the transfection control. **c** The corresponding changes of CFTR protein levels were monitored with SDS-PAGE and Western Blot analysis and normalized to the β-actin levels. Two individual samples (3 μg of total protein per lane) were tested for each treatment and the experiments were repeated twice. Error bars represent standard deviations (SD). Significant changes (*P* < 0.05) are marked with an asterisk

Given that miRNA levels and function in cancer cell lines often differs from primary cells, we examined miR-200b’s impact on CFTR expression in primary human lung cells (NHBEC Normal *Human Bronchial Epithelial Cells*) obtained from 3 donors. As shown in Fig. 6a, miR-200b was significantly induced ~2.5-fold during hypoxia in NHBECs, while the *CFTR* mRNA reduced by about 50% during normoxia (*P* = 0.07) and less so during hypoxia (Fig. 6b). However, the smaller effect during hypoxia may be due to already very low *CFTR* mRNA basal levels in NHBECs (approximately 50 fold lower than in immortalized cells, Additional file 1: Figure S1A). Inhibition of miR-200b by antagomiR, however, significantly increased *CFTR* mRNA levels in both conditions (Fig. 6b), confirming the physiological relevance of results obtained in immortalized cell lines. Importantly, we were able to observe a significance decrease in CFTR protein levels with miR-200b overexpression during normoxia, and a decrease during hypoxia, though it was not significant (*P* = 0.08) in NHBECs (Fig. 6c). miR-200b antagomiR treatment resulted in a significant increase in CFTR protein levels during both normoxia and hypoxia and this supported by the changes in *CFTR* mRNA as well. Importantly, our results obtained in primary human lung cells confirmed that miR-200b regulates CFTR expression.

## Discussion

The regulation of *CFTR* expression appears to be tissue specific and understanding its regulation is important in potential therapies for cystic fibrosis (CF) given that many of the disease causing mutations result in lower expression of this critically vital gene. Transcriptional regulation of *CFTR* is complex and includes elements within the promoter and intronic enhancers (reviewed in [25]). It has also been established that miRNA networks regulate *CFTR* expression as well [5, 26].

Although the role of miRNAs in posttranscriptional gene regulation is clearly established, it is now becoming evident that recent studies have shown that specific alterations in miRNA expression occur in cystic fibrosis (reviewed in [27]). Moreover, differences in miRNA expression are also present in chronic obstructive pulmonary disease, asthma, lung inflammation, and in smoke exposure in humans [28], suggesting that miRNA network changes can potentially influence disease pathogenesis. For CF, this is illustrated for the ΔF508 *CFTR* mutation, the most common mutation in CF, by studies that show that there is increased expression of miR-145, miR-223, and miR-494 in vivo in the bronchial epithelium of ΔF508 patients and this correlated with decreased CFTR expression [25, 29]. Furthermore, the introduction of a miRNA site through a mutation has been shown to increase the affinity of a miRNA that in vitro lowers the expression of the CFTR protein [30].

Using transcriptomic mRNA and miRNA-array-based experiments in colonic epithelial cells, Guimbellot and colleagues demonstrated that a number of genes were up- or down-regulated during hypoxia and *CFTR* was one of those genes that was downregulated [4]. It has also been reported that HIF-1 expression decreases CFTR expression in intestinal epithelium [18], suggesting that transcriptional regulation controls CFTR repression during hypoxia. The goal of the present study was to determine the relative contribution of miRNA-mediated post-transcriptional mechanisms as well.

Our in silico predictions indicated that miR-200b and miR-200c were putative candidates for *CFTR* posttranscriptional regulation. Using a hypoxia time course, we show that *CFTR* mRNA expression decreased after 8 h in both human airway epithelial cell lines, whereas HIF-1α protein expression was elevated as early as 2 h. Interestingly, the CFTR protein levels were not dramatically lower until 12 h, suggesting that more than just HIF-1 suppression of *CFTR* expression was occurring. To test for potential miRNA effects on *CFTR* expression, we utilized luciferase reporter constructs containing either the human *CFTR* 5′UTR, the 3′UTR or both and, in conjunction with chemical mimics of hypoxia that stabilized HIF-1 protein expression. We found that the 3′UTR was the more important region for suppression of expression, but that both regions were important and additive in their effects.

To differentiate between miR-200b and miR-200c, we found that miR-200b was elevated in both cell lines during hypoxia, whereas miR-200c was not. Furthermore, in Guimbellot et al.’s analysis of HT29 colonic epithelial cells, they identified 28 miRNAs that were upregulated during hypoxia, and miR-200b was one of them [4]. This suggested that miR-200b could have effects on *CFTR* mRNAs in more than just airway epithelia. Using the 3′UTR *CFTR* luciferase constructs we also demonstrated that miR-200b had a direct effect on luciferase expression, and this clearly established a direct effect on *CFTR* message levels. Final support for the role of miR-200b comes from the negative and positive effects of miR-200b mimics and antagomirs on *CFTR* expression changes, including the results in the primary airway cells. Taken together, the results suggest that during low oxygen conditions which could occur in various lung pathologies, miR-200b is upregulated and has a direct inhibitory effect on *CFTR* message and protein expression in human airway epithelial cells.

## Conclusions

**Fig. 7:**
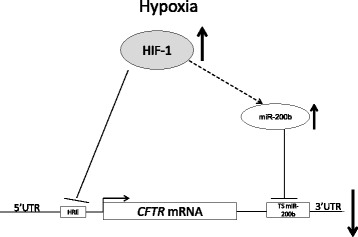
Model for negative regulation of *CFTR* expression during hypoxia by *HIF-1* and miR-200b. During hypoxia, *HIF-1* activity is induced and HIF-1 binds to the hypoxia response element (HRE) sequence located in *CFTR* 5′UTR and has been reported to decrease CFTR expression (Zheng et al. [18]). Our studies show that HIF-1 induces miR-200b expression and binds to the target sequence (TS miR-200b) located at the 3′UTR of *CFTR* mRNA, which further decreases *CFTR* mRNA and protein expression

In summary, our studies suggest that the HIF-1 dependent physiological changes in miR-200b levels in human airway epithelia under hypoxia contribute directly to CFTR downregulation during hypoxia. Hence, this results complement previous studies indicating HIF-1’s direct transcriptional effects on downregulation of *CFTR* with an additive post-transcriptional mechanism that involves a hypoxia-induced miRNA (Fig. 7). Furthermore, stabilization of CFTR protein levels during hypoxia through inhibition of miR-200b’s actions may provide a novel therapeutic opportunity for increasing CFTR expressions levels during various lung pathologies.

## Additional file

Additional file 1: Figure S1. Endogenous levels of miR-200b in NHBEC, Calu3 and 16HBE14o- cells. (A) Comparison of *CFTR* mRNA (white) and miR-200b (grey) relative levels between NHBEC (primary cells), Calu3 and 16HBE14o- cells during normoxic conditions. *CFTR* mRNA levels from 2 independent experiments (*n* = 8) are plotted normalized to 18S rRNA levels and expressed as a fold change over the NHBEC levels. miR-200b levels from 2 independent experiments (*n* = 8) are plotted normalized to *RNU48* levels and expressed as a fold change over the NHBEC levels. (B) NHBEC, Calu3 and 16HBE14o- cells were transfected with miR-200b antagomir (left) or mimic (right) and the miRNA levels were monitored in qRT-PCR experiments. miR-200b levels from 2 independent experiments (*n* = 8) are plotted normalized to *RNU48* levels and expressed as a fold change over the transfection control. Error bars represent standard deviations (SD). Significant changes (*P* < 0.05) are marked with an asterisk. (PDF 348 kb)

## Abbreviations

CFTR: Cystic fibrosis transmembrane conductance regulator
HIF: Hypoxia inducible factor
miRNA: microRNA
