# Supplementary figures and images for: miR-200b downregulates CFTR during hypoxia in human lung epithelial cells
Source: Cell Mol Biol Lett. 2017 Nov 15;22:23. doi: 10.1186/s11658-017-0054-0 (PMC5688675; doi:10.1186/s11658-017-0054-0)

**A**

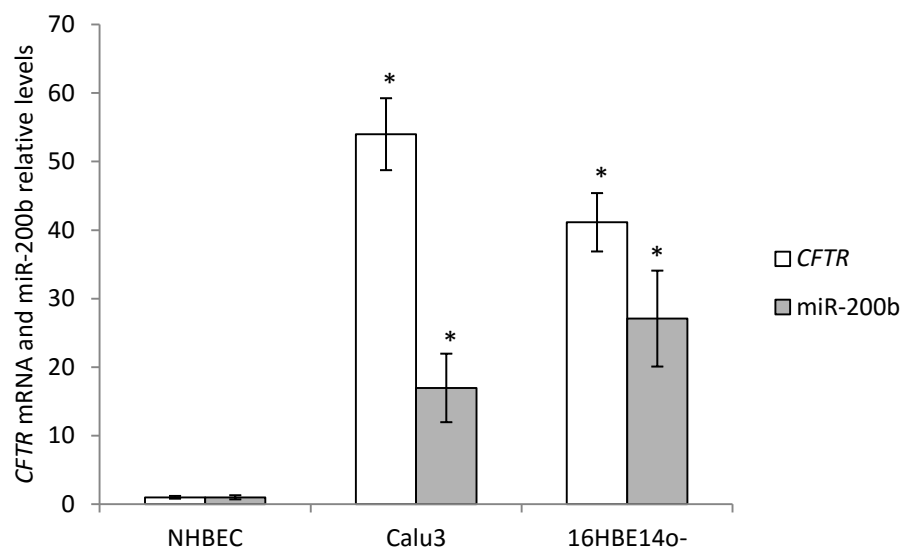

**B**

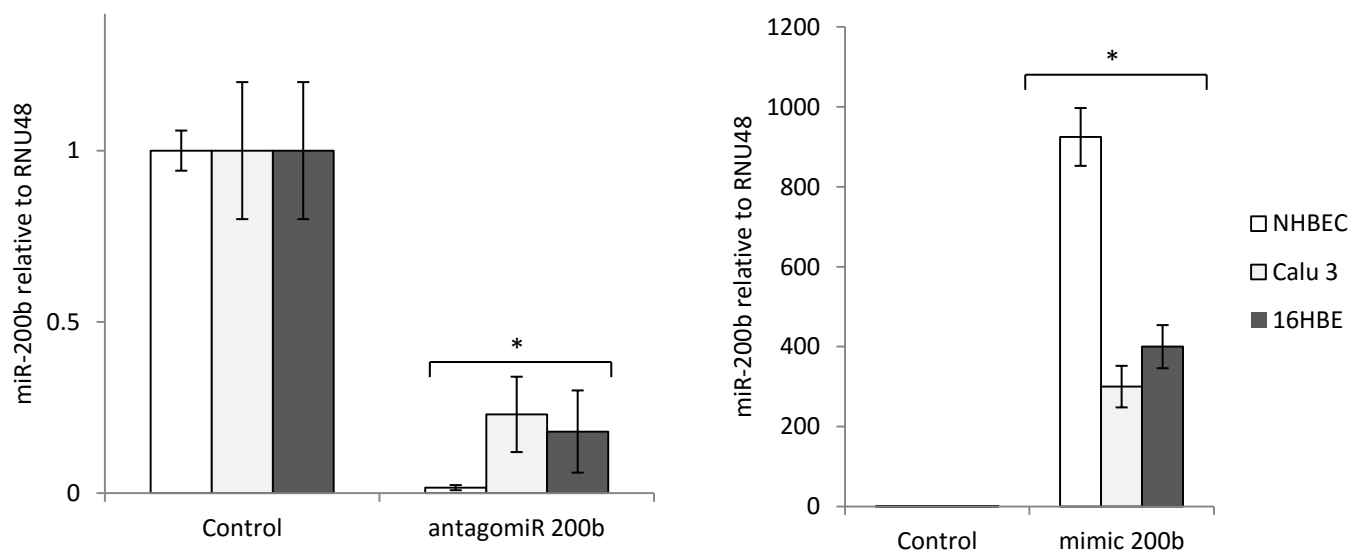

Supplement: Additional file 1: Figure S1. — Endogenous levels of miR-200b in NHBEC, Calu3 and 16HBE14o- cells. (A) Comparison of CFTR mRNA (white) and miR-200b (grey) relative levels between NHBEC (primary cells), Calu3 and 16HBE14o- cells during normoxic conditions. CFTR mRNA levels from 2 independent experiments (n = 8) are plotted normalized to 18S rRNA levels and expressed as a fold change over the NHBEC levels. miR-200b levels from 2 independent experiments (n = 8) are plotted normalized to RNU48 levels and expressed as a fold change over the NHBEC levels. (B) NHBEC, Calu3 and 16HBE14o- cells were transfected with miR-200b antagomir (left) or mimic (right) and the miRNA levels were monitored in qRT-PCR experiments. miR-200b levels from 2 independent experiments (n = 8) are plotted normalized to RNU48 levels and expressed as a fold change over the transfection control. Error bars represent standard deviations (SD). Significant changes (P < 0.05) are marked with an asterisk. (PDF 348 kb) [file 11658_2017_54_MOESM1_ESM.pdf]
